# Application of VA-ECMO combined with IABP in a patient with cardiac arrest following fulminant myocarditis

**DOI:** 10.1186/s13019-026-03851-1

**Published:** 2026-02-04

**Authors:** Yixin He, Ying Yang

**Affiliations:** Emergency Department, Peking University Shenzhen Hospital, Shenzhen, Guangdong Province China

**Keywords:** Fulminant myocarditis, VA-ECMO, IABP, Mechanical circulatory support

## Abstract

**Background:**

Fulminant myocarditis is a severe cardiac condition characterized by rapid progression and high mortality. In recent years, advancements in medical technology, particularly the application of mechanical circulatory support, have significantly improved patient outcomes.

**Case presentation:**

A 36-year-old male patient was admitted with a history of “fever for 5 days and chest tightness and pain for 2 days”.He was diagnosed with acute myocarditis and subsequently experienced cardiac arrest. After resuscitation, he was transferred to the medical ICU, where he received VA-ECMO and IABP support therapy, eventually recovering and being discharged.

**Conclusion:**

Early recognition, timely intervention, and mechanical circulatory support are crucial in the treatment of fulminant myocarditis, significantly enhancing patient survival rates.

## Background

Acute myocarditis is an inflammatory disease of the myocardium caused by various etiologies, with a wide range of clinical presentations, from asymptomatic to cardiogenic shock and even sudden death [1, 2]. Fulminant myocarditis is a severe form of acute myocarditis characterized by rapid progression and high mortality [3, 4]. In recent years, mechanical circulatory support devices such as VA-ECMO and IABP have played a crucial role in the treatment of fulminant myocarditis. This report presents a case of fulminant myocarditis complicated by cardiac arrest, where early initiation of VA-ECMO combined with IABP led to successful recovery, providing new insights into the individualized treatment of severe myocarditis.

## Case presentation

**Fig. 1:**
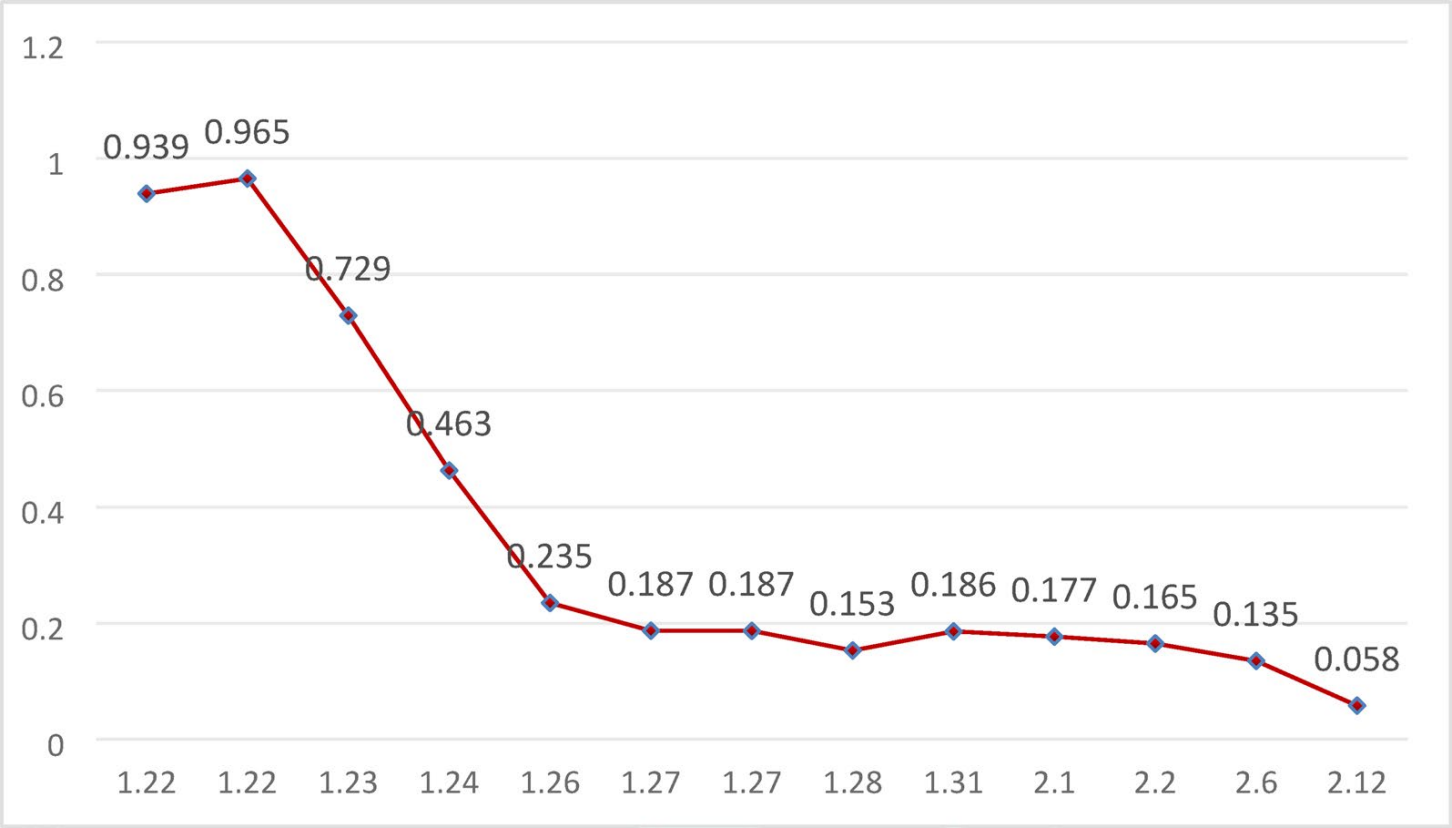
hs-cTnT change curve (reference value: < 0.014ng/ml)

**Fig. 2:**
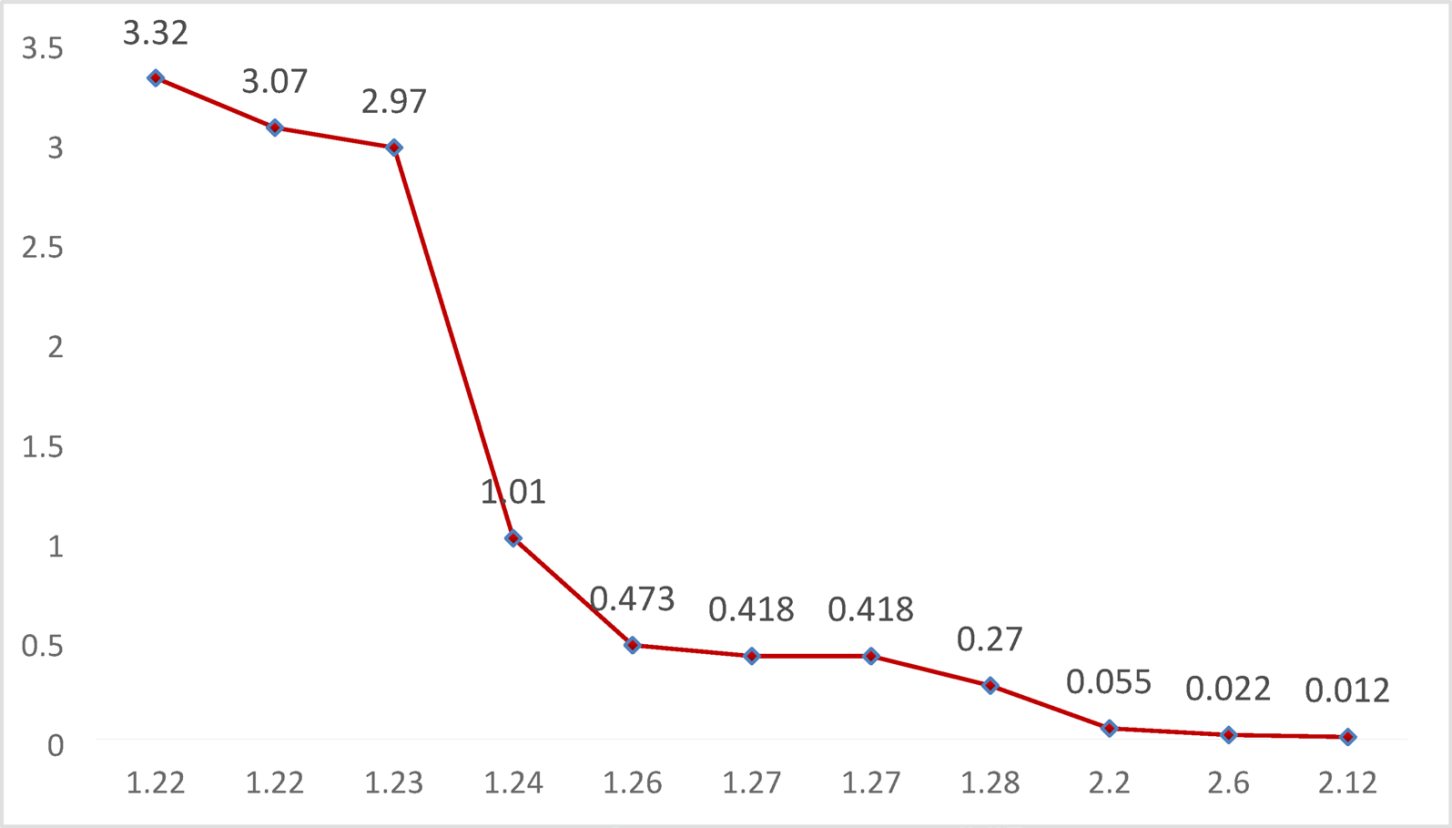
hs-cTnI change curve (reference value: < 0.034ng/ml)

year-old male patient was admitted on January 22, 2025, with complaints of “fever for 5 days and chest tightness and pain for 2 days.” Five days prior to admission, the patient developed a fever after exposure to cold weather, which did not improve with over-the-counter cold medication. Two days before admission, he experienced intermittent chest tightness accompanied by fever, with a maximum body temperature of 38.0 °C. He sought treatment at our emergency department, where laboratory results indicated troponin I levels of 3.320 ng/mL and troponin T levels of 0.939 ng/mL (the dynamic trends are shown in Fig. 1 and Fig. 2), suggesting acute myocarditis. An electrocardiogram showed sinus tachycardia, left anterior fascicular block, and low QRS voltage. He was transferred to the emergency resuscitation room for further diagnosis and treatment. Vital signs were as follows: temperature 36.5 °C, heart rate 104 bpm, respiratory rate 16 bpm, blood pressure 96/68 mmHg, and SpO₂ 100%. Physical examination revealed that the patient was conscious, with no cold or clammy skin, and no pallor or cyanosis of the oral mucosa.Echocardiography revealed diffuse ventricular wall motion abnormalities, slightly thickened interventricular septum, reduced left ventricular systolic function, and a small pericardial effusion.The SCAI shock stage was classified as Stage A. After consultation with a cardiologist, the patient was transferred to the cardiac intensive care unit (ICU) later that afternoon.Vital signs upon leaving the emergency room: Temperature 36.5 °C, heart rate 107 bpm, respiratory rate 16 bpm, blood pressure 99/71 mmHg, SpO₂ 98%; SCAI shock stage A.Laboratory findings: NT-proBNP: 25,219 pg/mL; Cardiac enzymes: lactate dehydrogenase (LDH) 453 U/L, creatine kinase (CK) 329 U/L, creatine kinase MB isoenzyme activity (CK-MB) 32.2 U/L; Liver function: alanine aminotransferase (ALT) 133 U/L, total bilirubin(TB) 13.3 µmol/L; D-dimer: 1.42 mg/L FEU; No significant abnormalities in coagulation profile, renal function, or electrolytes.However, during the transfer, he experienced cardiac arrest. Immediate bedside cardiac compression and administration of vasopressors were initiated. After approximately 10 min of cardiopulmonary resuscitation, the patient regained spontaneous heart rate and blood pressure, albeit with a rapid heart rate and low blood pressure, necessitating continuous norepinephrine infusion. Due to respiratory distress and poor oxygenation, he was provided with mask oxygen. Shortly thereafter, the patient became unconscious again, with agonal respiration (manifested as deep, nodding-headed breaths), and his heart rate dropped to 56 beats per minute, along with a decline in blood pressure and absence of palpable arterial pulses, indicating cardiac arrest. Immediate chest compressions and intravenous epinephrine were administered, resulting in the restoration of spontaneous rhythm. After the return of spontaneous circulation (ROSC) following cardiopulmonary resuscitation: Heart rate: 147 bpm, blood pressure: 86/41 mmHg; Arterial blood gas analysis: pH 7.23, PO₂ 75 mmHg, PCO₂ 39 mmHg, lactate 8.2 mmol/L; SCAI shock stage C.Epinephrine and norepinephrine infusions were continued to maintain heart rate and blood pressure. After discussing the condition with the family, the patient was transferred to the medical ICU for further resuscitative efforts. To ensure safety during transport, anesthesiologists were urgently requested to perform bedside tracheal intubation and mechanical ventilation. After intubation, the patient was connected to a transport ventilator, and continuous infusions of epinephrine and norepinephrine were maintained to support heart rate and blood pressure.Subsequently, the patient was transferred to the medical ICU for further monitoring and treatment.

**Fig. 3:**
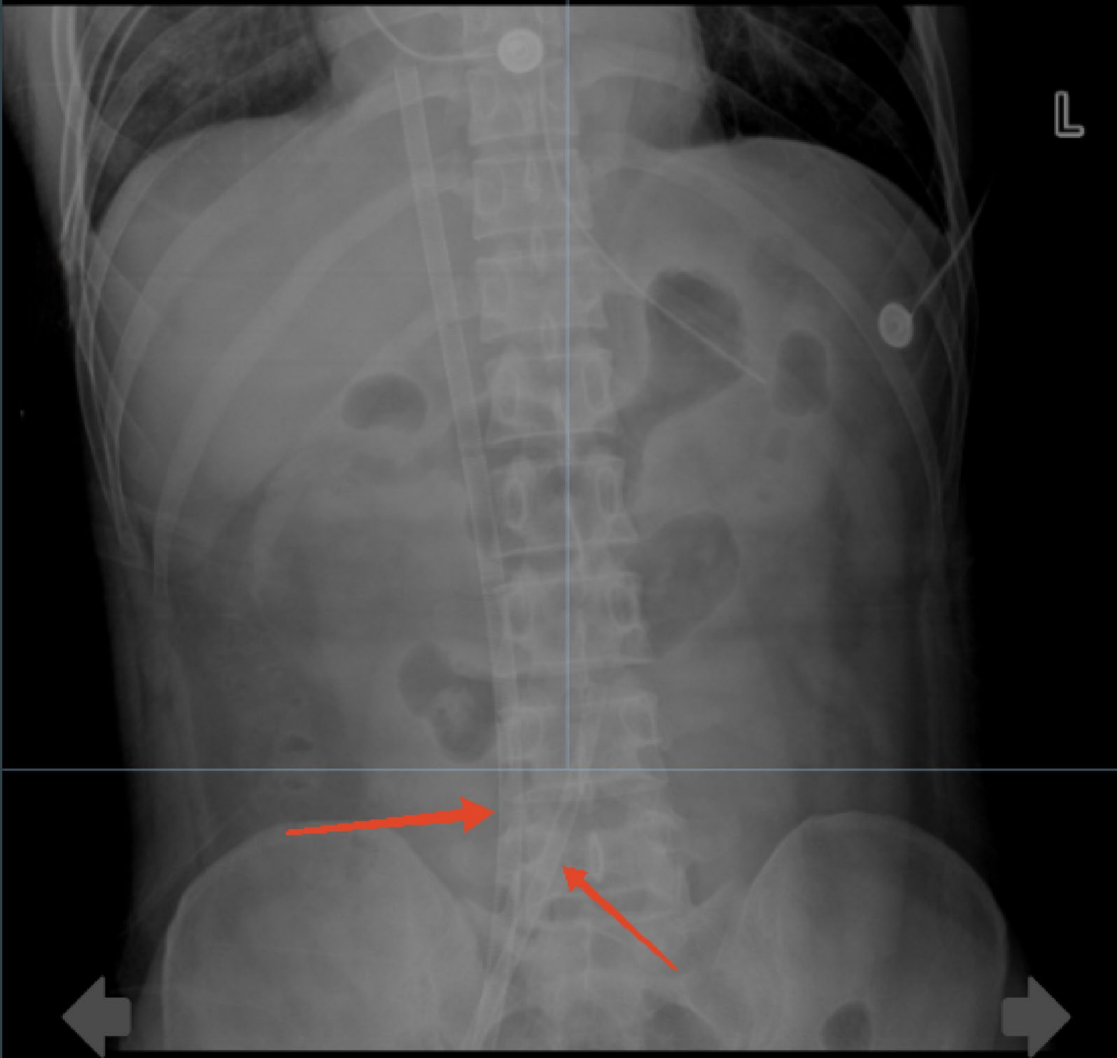
VA-ECMO tube

**Fig. 4:**
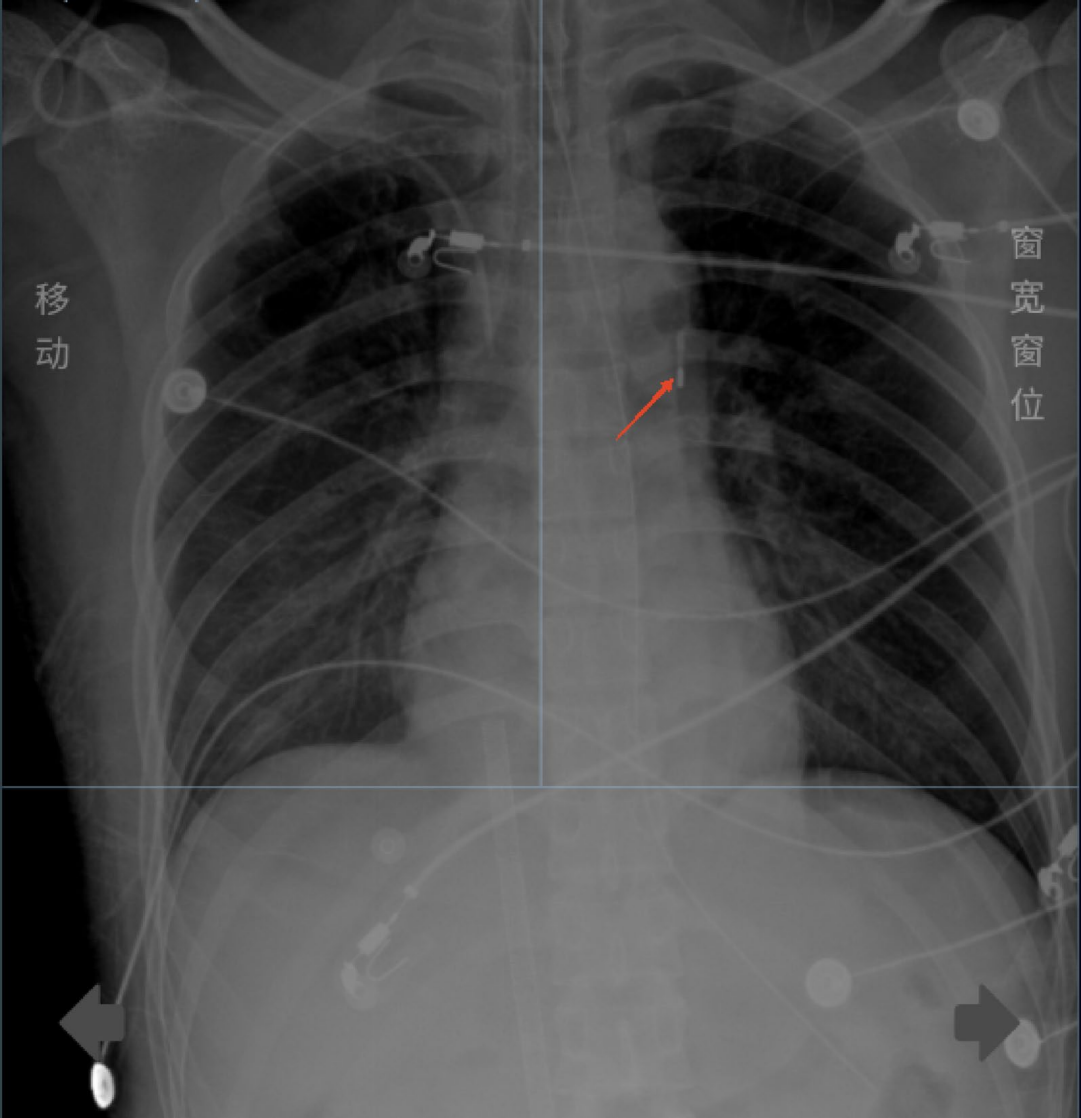
IABP balloon

Upon arrival at the medical ICU, immediate hemodynamic and critical care ultrasound assessments were conducted, revealing diffuse weakening of cardiac contraction, extremely low VTI and CO, and highly unstable hemodynamics, necessitating high-dose vasopressors to maintain blood pressure. The patient exhibited signs of cardiogenic shock, and considering his clinical history, fulminant myocarditis was suspected. Given the cardiogenic shock, the patient was deemed eligible for mechanical life support. After informing the family about the necessity and risks, VA-ECMO support was initiated on the day of admission (Fig. 3), followed by the addition of IABP therapy on January 23 (Fig. 4). During this period, the patient’s cardiopulmonary function, volume status, and brain oxygenation were closely monitored to ensure adequate cerebral and organ perfusion. Hypothermia therapy for brain protection was implemented once hemodynamics stabilized. The patient’s cardiac function subsequently improved significantly, allowing for the discontinuation of VA-ECMO on January 26.On January 26th, the indications for ECMO weaning were evaluated. Beginning at 9:30 AM, the ECMO flow was gradually reduced, reaching 1.6 L/min. The patient was assessed every 20 min. After the flow rate was decreased to 1.5 L/min, the following parameters were recorded: Cardiac monitor: Heart rate 63 bpm, MAP 80 mmHg, SpO₂100%; Arterial blood gas: pH 7.48, PCO₂ 32 mmHg, PO₂ 380 mmHg, lactate 1.5 mmol/L, P/F ratio 380 mmHg; Bedside cardiac ultrasound: EF 60%−70%, VTI 19 cm, CO 4 L/min.All parameters were satisfactory and met the criteria for ECMO decannulation. The ECMO decannulation procedure was performed from 11:30 AM to 11:45 AM. The wound was compression bandaged with an elastic bandage. The lower limb showed no significant mottling or signs of ischemia, and the dorsalis pedis pulse was palpable. The procedure was uneventful, with an estimated blood loss of 10 mL. Close monitoring of the dorsalis pedis pulse and circulatory status was maintained.Patient assessment 30–60 min after decannulation revealed: Cardiac monitor: Heart rate 69 bpm, MAP 87 mmHg, SpO₂ 100%; Arterial blood gas: pH 7.49, PCO₂ 33 mmHg, PO₂ 168 mmHg, lactate 1.9 mmol/L, P/F ratio 373 mmHg; Bedside cardiac ultrasound: EF 60%−70%, VTI 19.27 cm, CO 4.09 L/min.Subsequently, IABP support was withdrawn on January 27. Other treatments included immunomodulation with high-dose corticosteroids and immunoglobulins to reduce inflammation and edema and alleviate clinical symptoms; antiviral therapy with oseltamivir, considering the patient’s prior upper respiratory infection; and vasopressor support, which was gradually weaned off after the initiation of mechanical circulatory support, maintaining a mean arterial pressure (MAP) above 65 mmHg. Pulmonary management involved protective ventilation strategies to avoid lung injury, along with positive pressure support to reduce left ventricular preload and afterload, leading to successful extubation on January 29. For infection control, piperacillin-tazobactam was initially administered, but due to persistent fever, the regimen was empirically adjusted to vancomycin combined with meropenem on January 27. Sputum culture on February 2 identified sensitive Klebsiella pneumoniae and Candida. After treatment, the patient’s vital signs stabilized, and he no longer required advanced life support. He was transferred to the cardiac ICU on February 5, then to the general cardiac ward on February 6 for further rehabilitation, and was discharged on February 12.

## Discussion

Fulminant myocarditis is a severe cardiac condition with rapid clinical deterioration, often leading to life-threatening complications such as cardiac arrest [5]. From an emergency medicine perspective, timely and accurate risk assessment, particularly for cardiac arrest, is paramount in managing patients with fulminant myocarditis.

In this case, the patient received early high-dose glucocorticoids combined with immunoglobulin for immunomodulatory therapy immediately after transfer to the ICU. The pathophysiological rationale for this approach is that the early stage of fulminant myocarditis is essentially an innate immunity-dominated inflammatory storm. Glucocorticoids effectively promote the polarization of macrophages toward the anti-inflammatory M2 phenotype and suppress multiple inflammatory mediators, thereby rapidly creating a stable internal environment for myocardial repair.

ECMO is an extracorporeal life support technique that provides oxygenation and hemodynamic support by partially or fully replacing the patient’s cardiopulmonary function [6, 7]. IABP, on the other hand, reduces cardiac afterload and increases coronary blood flow by periodic inflation and deflation of an intra-aortic balloon, thereby improving myocardial perfusion. Both devices have demonstrated significant efficacy in the resuscitation of cardiac arrest and heart failure [8, 9]. Recent studies have shown that the combination of ECMO and IABP can synergistically improve left ventricular unloading and significantly reduce mortality compared with single instrument support [10–12]. With the widespread adoption of percutaneous cannulation techniques, the early application of these devices in emergency departments and ICUs is becoming a trend, although strategies to mitigate complications such as bleeding and limb ischemia still require optimization.

This patient presented with a typical triad in the emergency department: a young male, “flu-like” prodromal symptoms, and significantly elevated troponin levels, aligning with the red alert criteria of the 2021 International Myocarditis Consensus [13]. The rapid completion of bedside ultrasound assessment and initiation of urgent cardiology consultation in the emergency department exemplifies the advantages of managing “time-sensitive” conditions. The patient experienced cardiac arrest twice after being transferred, indicating that the disease was in the “electrical storm” phase. The timely initiation of VA-ECMO and IABP played a crucial role in this patient’s treatment. VA-ECMO provided extracorporeal circulatory support, ensuring systemic oxygenation and perfusion, while IABP reduced cardiac afterload and improved coronary blood flow. The combined use of these devices bought valuable time for the patient’s cardiac recovery. In addition to mechanical circulatory support, comprehensive treatment measures such as immunomodulation, antiviral therapy, and infection control were also implemented. The combination of these strategies effectively controlled the patient’s condition and facilitated recovery. With active treatment, the patient’s cardiac function gradually improved, and he was successfully weaned off mechanical circulatory support.

The question of whether early IABP implantation would have been more beneficial represents a highly valuable clinical decision point. For this patient, we analyzed the potential advantages and disadvantages of early IABP implantation as follows:

1. Potential Benefits of Early IABP Implantation

1. Stabilizing Electrical Storm and Preventing Recurrent Arrest: After the first cardiac arrest, the patient remained in an “electrical storm” phase with extreme myocardial instability. IABP improves the balance between myocardial oxygen supply and demand by increasing diastolic coronary perfusion pressure and oxygen supply while reducing left ventricular afterload and oxygen consumption during systole. This stabilization effect may help calm the irritated and damaged myocardium, potentially reducing the risk of recurrent malignant arrhythmias and cardiac arrest. If IABP had been implanted immediately after the first resuscitation, the second cardiac arrest during transfer to the ICU might have been avoided.
2. Creating More Stable Conditions for VA-ECMO Placement: Performing VA-ECMO cannulation under extremely unstable hemodynamic conditions carries high risks. Early IABP support could provide partial circulatory assistance, potentially transiently improving blood pressure and coronary blood flow, thereby creating a more stable “time window” and “clinical window” for subsequent successful VA-ECMO establishment.
3. Early Left Ventricular Unloading to Prevent VA-ECMO-Induced Pulmonary Edema: While VA-ECMO provides circulatory support, it simultaneously increases left ventricular afterload, potentially raising left ventricular wall tension and triggering or exacerbating pulmonary edema. Early combination with IABP could initiate left ventricular unloading concurrently with or even before VA-ECMO initiation, representing a “proactive prevention” strategy rather than “reactive treatment,” which might be more beneficial for lung protection.

2. Clinical Considerations for Delayed IABP Combination Until After VA-ECMO

Despite the theoretical advantages of early implantation, the decision in this case to combine IABP only on the day after VA-ECMO initiation was likely based on the following more cautious considerations:

1. Extreme Criticality of the Condition and Priority of Support Intensity: After the first cardiac arrest, although spontaneous circulation returned, the patient remained in severe cardiogenic shock, requiring high-dose vasopressors. The most urgent need at that moment was rapid establishment of powerful, comprehensive circulatory and oxygenation support. VA-ECMO is far superior to IABP in this regard. Therefore, concentrating all resources on establishing VA-ECMO with the highest efficiency was the most direct and effective life-saving measure. When “saving life” is the primary goal, prioritizing the modality with the strongest support capacity is justified.
2. Limitations of IABP Support in Pure Myocarditis: IABP itself does not directly provide oxygenation, and its circulatory support capacity is limited. For this case of fulminant myocarditis with diffusely impaired cardiac contraction and extremely low cardiac output, IABP alone might have been insufficient to maintain effective end-organ perfusion and could not replace the decisive role of VA-ECMO.
3. Balancing Procedural Risks and Benefits: Following cardiac arrest resuscitation, with potential coagulation disorders, emergency IABP implantation also carries risks such as difficult puncture, bleeding, and limb ischemia. Performing two invasive procedures (IABP followed by VA-ECMO) in such an unstable state could pose a cumulative risk higher than first establishing stable VA-ECMO life support and then evaluating and implanting IABP after the condition has somewhat stabilized.

In addition to VA-ECMO and IABP, percutaneous micro-axial flow pumps (such as the Impella series) have gained significant attention in recent years as another powerful mechanical circulatory support device for the treatment of acute myocarditis complicated by cardiogenic shock.

The Impella is a micro-axial flow pump implanted into the left ventricle through the aortic valve. Its working principle involves directly pumping oxygenated blood from the left ventricle into the ascending aorta, thereby providing powerful direct left ventricular unloading and circulatory support.

Advantages of Impella:

1. Pure and Powerful Left Ventricular Unloading: Unlike IABP, which primarily reduces afterload, the Impella actively aspirates blood from the left ventricle, significantly reducing the left ventricular end-diastolic pressure (LVEDP) and ventricular wall tension, achieving true “ventricular unloading.” This offers clear physiological benefits for alleviating myocardial edema, reducing myocardial oxygen consumption, and promoting myocardial recovery.
2. Increased Cardiac Output: The Impella can provide flow rates up to 2.5 L/min to 5.0 L/min, offering far greater circulatory support capability than IABP. For some patients who are not in extremis, it can even be used alone as a “bridge to recovery” support modality.

Rationale for Ventricular Unloading in Acute Myocarditis:

The core pathophysiology of fulminant myocarditis involves diffuse myocardial inflammation, edema, and necrosis, leading to severely impaired myocardial contractility, cardiac dilation, and the initiation of a vicious cycle:

- A sharp decrease in cardiac output leads to hypotension and inadequate organ perfusion.
- A significant increase in left ventricular end-diastolic pressure causes pulmonary venous congestion, leading to pulmonary edema.
- Cardiac dilation and increased wall tension raise myocardial oxygen demand while simultaneously compressing the subendocardial coronary arteries, reducing myocardial blood supply, thereby exacerbating myocardial ischemia and injury.

Therefore, the goal of “ventricular unloading” in the treatment of fulminant myocarditis is to break this vicious cycle. By reducing the pressure and volume load on the left ventricle, the following can be achieved:

1. Reduce the myocardial workload, creating a “resting” opportunity for myocardial recovery.
2. Improve subendocardial myocardial perfusion, rescuing jeopardized myocardial cells.
3. Effectively alleviate pulmonary edema and improve oxygenation.

Considerations and Decision-Making Rationale for Not Using Impella in This Case:

Despite the theoretical advantages of Impella in left ventricular unloading, our emergency decision-making in this case prioritized the VA-ECMO combined with IABP strategy, primarily based on the following considerations:

1. Need for Comprehensive Support Level: The patient rapidly developed cardiac and respiratory arrest after admission, accompanied by “poor oxygenation,” indicating concurrent extreme circulatory and respiratory failure. VA-ECMO (veno-arterial mode) can simultaneously provide powerful cardiopulmonary replacement support, addressing both core issues of oxygenation and circulation. In contrast, Impella provides only circulatory support and cannot improve pulmonary function. The subsequent appearance of diffuse B-lines and pulmonary consolidation in both lungs in this case further underscored the critical importance of concurrent respiratory support.
2. Potential for Biventricular Involvement: Fulminant myocarditis often affects both ventricles simultaneously. VA-ECMO, via right atrial drainage, can concurrently reduce the right heart load. A purely left ventricular assist device like Impella offers limited support for severe right heart failure and might even exacerbate the right ventricular burden due to increased right ventricular output.
3. Rapid Clinical Deterioration and Decision-Making Efficiency: The patient suffered cardiac arrest during transfer, with the condition manifesting as an “electrical storm,” necessitating the fastest possible establishment of the most reliable life support system. Opting for VA-ECMO avoided the potential risk of missing the critical rescue window should Impella implantation encounter difficulties or delays.
4. Flexibility for Subsequent Combined Therapy: After establishing VA-ECMO as a stable life support platform, we could more deliberately assess the patient for persistent left ventricular dilation or pulmonary edema. Upon identifying that VA-ECMO alone might increase left ventricular afterload, IABP was subsequently combined for left ventricular unloading. This formed a stepped, problem-oriented, precise treatment strategy. IABP, being a more widely available and technically mature technology in this context, when combined with VA-ECMO, effectively achieved the goals of reducing afterload and improving coronary blood flow.

The mechanism of cardiac arrest in fulminant myocarditis differs from that in coronary artery disease, primarily stemming from acute inflammation-mediated myocardial electrical instability rather than coronary ischemia [14, 15]. In this patient, cardiac arrest during transfer may have been related to mechanical stimulation triggering a sympathetic storm [16], with positional changes causing mechanical stretch of the heart, activating transient receptor potential (TRP) channels in the inflamed myocardium, and triggering malignant arrhythmias [17, 18].

The successful outcome of this case was attributed to the seamless collaboration between the emergency department, cardiology department, and ICU, although there is still room for improvement, such as improving the right heart catheterization examination to evaluate cardiopulmonary function at an early stage and developing a risk prediction model for fulminant myocarditis.The successful treatment of this patient demonstrates that early recognition, timely intervention, and mechanical circulatory support are key to managing fulminant myocarditis. With ongoing advancements in medical technology, it is anticipated that more effective treatments will be available in the future, further enhancing the success rate of treating fulminant myocarditis.

## Data Availability

The authors stated that all the data and materials were true and available in this study.
